# Preventive Behaviors During the COVID-19 Pandemic: Associations With Perceived Behavioral Control, Attitudes, and Subjective Norm

**DOI:** 10.3389/fpubh.2021.662835

**Published:** 2021-05-07

**Authors:** Damaris Aschwanden, Jason E. Strickhouser, Amanda A. Sesker, Ji Hyun Lee, Martina Luchetti, Antonio Terracciano, Angelina R. Sutin

**Affiliations:** ^1^Department of Geriatrics, Florida State University College of Medicine, Tallahassee, FL, United States; ^2^Department of Behavioral Sciences and Social Medicine, Florida State University College of Medicine, Tallahassee, FL, United States

**Keywords:** COVID-19, Theory of Planned Behavior, preventive health behaviors, older adults, perceived behavioral control, attitudes, subjective norm

## Abstract

**Background:** The coronavirus disease 2019 (COVID-19) is a highly contagious and potentially fatal infectious disease that has swept the globe. To reduce the spread, it is important to engage in preventive behaviors recommended by health authorities, such as washing your hands, wearing a face mask, and social distancing.

**Aim:** In the present study, we draw from the Theory of Planned Behavior (TPB) to examine the associations between perceived behavioral control, attitudes, and subjective norm and whether people engage in eight different preventive behaviors.

**Methods:** For each of the preventive behaviors (washing hands; using hand sanitizer; not touching your face; social distancing; wearing a face mask; disinfecting surfaces; coughing in your elbow; staying home if sick), we conducted separate logistic regressions predicting whether the participants (*N* = 2,256; age range = 1898 years) reported engaging in the behavior from their perceived behavioral control, attitudes, and subjective norm.

**Results:** We found that perceived behavioral control, attitudes, and subjective norm had independent significant associations with each preventive behavior. Moderation analyses revealed that for most behaviors the associations with perceived behavioral control were stronger for older adults than for younger adults.

**Limitation:** The present study was cross-sectional; future longitudinal studies and interventions are needed to disentangle directionality.

**Conclusion:** Our findings suggest several ways to increase adherence to health behaviors that reduce the spread of coronavirus and other infectious diseases.

## Introduction

The coronavirus disease 2019 (COVID-19) is a highly contagious and potentially fatal infectious disease that has swept the globe. On January 30, 2020, the World Health Organization (WHO) declared COVID-19 a Public Health Emergency of International Concern (1), and on March 11, 2020 the WHO declared it a global pandemic (2). Recent Emergency Use Authorization of COVID-19 vaccines and approval of an anti-viral drug (remdesivir) provide a pathway forward (3), but the journey to end the pandemic is not over yet. As it wears on, a significant way to prevent the disease is still to avoid exposure to the virus by engaging in preventive behaviors. The Centers for Disease Control and Prevention (CDC) recommends preventive behaviors such as washing hands, using hand sanitizer, wearing a face mask, and social distancing to reduce the risk of contracting or spreading the virus (4), even after one has been fully vaccinated against COVID-19 (5). It is thus still of utmost importance that as many people as possible engage in them. However, there are individual differences in how strictly people adhere to these guidelines. It is crucial to understand why some individuals are following the recommendations and others are not, as a first step to increase adherence.

The Theory of Planned Behavior (TPB) (6) is one model that aims to explain how individual factors are associated with engaging in specific behaviors. The TPB proposes that (a) perceptions of control over engaging in a behavior, (b) positive attitudes toward a behavior, and (c) subjective norms to perform that behavior predict behavioral intentions, and that those intentions subsequently predict whether an individual engages in the specific behavior. The perceived behavioral control indicates a person's perception of how easy or difficult it is to perform a behavior (7, 8). The attitude toward a behavior indicates a person's general assessment of whether a particular behavior is either positively or negatively valued (9). The subjective norm refers to a person's perceived social pressure to or not to perform a particular behavior (10). While the perceived behavioral control is the main component of the nonvolitional process, the attitude and the subjective norm are components of the volitional process (6). In the context of the COVID-19 pandemic, each component may be a crucial determinant in the prediction of whether people engage in preventive behaviors. For example, people may be more likely to use hand sanitizer if they think this precaution is easy to do (high perceived control), effective (positive attitude toward hand sanitizer), and everyone else does it (high perceived social pressure). In contrast, people may be less likely to stay 6 feet away from others if they think this precaution is difficult to do (low perceived control), ineffective (negative attitude toward social distancing), and only a few other people do it (low perceived social pressure). The present study aims to examine whether perceived control, attitudes, and subjective norms of preventive behaviors were associated with engagement in those behaviors. Understanding how the three core components of the TPB play out during the COVID-19 pandemic may offer valuable insights for public health organizations that aim to increase the number of people engaging in preventive behaviors during this pandemic and in future infectious disease outbreaks.

The positive associations among perceived behavioral control, attitudes, subjective norms, and behavioral intention have long been tested and demonstrated in meta-analytic work on a broad range of health behaviors that provide empirical support for the conceptual justification of the TPB (6). For example, the TPB predicts healthy food choices (11), treatment adherence in chronic illness (12), condom use (13), alcohol consumption (14), and sun-protective behavior (15). Moreover, a meta-analysis of 237 prospective studies found that on average, the TPB predicted 19.3% of the variance in health behaviors (16).

Less is known about the predictive power of the TPB and preventive health behaviors in response to large-scale disease outbreaks. One quasi-experiment found that the TPB predicted students' mask-wearing intentions during a hypothetical future pandemic (17). However, it is unclear to what degree this research applies to the real context of a pandemic. A study conducted during the outbreak of severe acute respiratory syndrome (SARS) in 2003 found that the TPB predicted engaging in preventive behaviors in four small samples (18). The outcome, however, was a general score of preventive behaviors that did not distinguish between the different types of behaviors. That is, the outcome was a sum of 15 behaviors that assessed diverse topics such as cleaning (e.g., washing hands and clothes, disinfecting surfaces), isolating (e.g., avoid going out to eat, shopping, or shaking hands, wear a face mask), information gathering (e.g., news reports, healthcare workers, internet), and general health (e.g., diet, exercise, sleep). Thus, it is unclear from the Cheng and Ng study whether the associations apply to all the preventive behaviors they included or just a few.

Interest in and evidence for the association between the TPB and preventive health behaviors in the current COVID-19 pandemic has also been growing. For example, TPB predicted whether factory workers (19) and parents with children under the age of 18 years (20) in China intend to get a COVID-19 vaccination for themselves or their children, respectively. Furthermore, a study conducted in China found that attitude toward epidemic prevention affected individuals' intention to adopt epidemic prevention, while norms did not have a significant impact, and perceived feasibility to adopt epidemic prevention was found to be a barrier (21). Another study (22) suggests that perceived behavioral control, intentions, forms of planning, and maintenance self-efficacy are prominent predictors of COVID-19 preventive behaviors (a latent variable consisting of washing hands frequently, maintaining social distancing, practicing respiratory hygiene, and staying home if feeling unwell). In a study of adults with chronic conditions (23), subjective norm and perceived behavioral control were positively associated with intention to engage in preventive behaviors (a mean score of hand washing, avoiding touching one's face, staying home when sick, covering mouth/nose when coughing or sneezing, keeping physical distancing, use of face mask, and self-isolation). Although these studies provide important information on the perception of COVID-19 preventive behaviors, they focused on a composite score of such behaviors. Only a few studies focused on single preventive behaviors, although the number of studies is increasing. For instance, positive but not negative attitudes, perceived behavioral control, and subjective norm were significantly associated with social distancing (24). Moreover, perceived effectiveness of preventive measures was associated with higher compliance of using hand sanitizer and avoiding gatherings with people outside one's household (25).

The present study examines the association of perceived control, attitudes, and subjective norm with engagement in eight different preventive behaviors during the early days of the COVID-19 outbreak in the United States (i.e., March 1829, 2020), when Americans were asked by the White House to follow the 15 days to slow the spread coronavirus guidelines (26). These guidelines advised Americans to (a) stay home if they feel sick, (b) keep sick children at home, (c) isolate if someone from their household has tested positive for the coronavirus, (d) work or engage in schooling from home, (e) avoid social gathering of more than 10 people, (f) avoid going to bars and restaurants, (g) avoid travel, shopping trips, and social visits, (h) avoid visiting nursing homes or long-term care facilities, and (i) practice good hygiene. Older adults and persons with pre-existing conditions were further advised to stay home and avoid contact with others. At the end of March 2020, the White House extended these recommendations for an additional 30-day period. We analyze the associations separately for each behavior: (1) washing hands, (2) using hand sanitizer, (3) not touching your face, (4) social distancing, (5) wearing a face mask, (6) disinfecting surfaces, (7) coughing in your elbow, and (8) staying home if sick. We hypothesize that all three factors are significantly associated with engaging in each specific behavior. In exploratory analyses, we first examine whether the associations are moderated by participant age in years, because older adults are more vulnerable to complications of COVID-19 (27) and younger adults may feel less threatened by COVID-19 (28). Additionally, we examine whether the three factors interact in their association with behavior, as such interactions have been hypothesized in other behavioral contexts (29, 30). If they are present, they may be important for understanding engagement in preventive behaviors.

## Materials and Methods

### Participants and Procedure

The present study is part of a larger survey project (31,34). The original project aimed to investigate loneliness, personality, and health in late January 2020. In March 2020, when the coronavirus spread was declared a national emergency, participants from the original project were recontacted to complete a second assessment on responses to COVID-19. The data for the present analyses come from this assessment and were collected between March 18 and 29, 2020. The present study thus is cross-sectional. Participants from every U.S. state as well as Washington, D.C. and Puerto Rico were originally recruited and recontacted by Dynata (https://www.dynata.com/) and directed to a Qualtrics survey. Participants were recruited evenly across age groups (1819, 2029, 3039, 4049, 5059, 6069, and 70+), as well as 50% female and 20% African American. Of the original sample, *n* = 2,485 participants consented to complete our COVID-19 survey. We excluded 153 participants who did not provide complete information for the demographic covariates and a further 76 participants who did not respond to the variables of interest in this study. Thus, a total of 2,256 participants were included in our final sample. Material and procedures were reviewed and approved by the Institutional Review Board of Florida State University.

### Measures

#### Preventive Behaviors

We asked participants In general, what precautions are you taking to avoid catching the coronavirus? Check all that apply. The response options were based on recommendations on sickness prevention published by the CDC (4) as well as previous work on preventive behaviors to avoid catching influenza (35). They included eight behaviors: Wash your hands often, Use hand sanitizer, Avoid touching your eyes, nose, and mouth, Put physical distance between yourself and other people (social distancing), Wear a face mask, Clean and disinfect surfaces, Cough and sneeze in your elbow or tissues, and Stay home if you are sick. Each behavior was scored as yes = 1 or no = 0.

#### Perceived Behavioral Control

For each of the eight behaviors, we asked participants To reduce the spread of the coronavirus, how DIFFICULT is it for you to follow these recommendations?. Responses were assessed on a 5-point Likert-type scale (1 = Extremely easy, 2 = Somewhat easy, 3 = Neither easy nor difficult, 4 = Somewhat difficult, 5 = Extremely difficult). Before the analysis, the scales were reversed so that higher values indicated greater ease.

#### Attitudes Toward Behaviors

For each of the eight behaviors, we asked participants To reduce the spread of the coronavirus, how EFFECTIVE do you believe these recommendations are?. Responses were assessed on a 5-point Likert-type scale (1 = Extremely ineffective, 2 = Somewhat ineffective, 3 = Neither effective nor ineffective, 4 = Somewhat effective, and 5 = Extremely effective).

#### Perceived Subjective Norm

For each of the eight behaviors, we asked participants To reduce the spread of the coronavirus, how many OTHER PEOPLE do you think are following these recommendations?. Responses were assessed on a 5-point Likert-type scale (1 = No one, 2 = Few people, 3 = Some people, 4 = Most people, and 5 = Everyone).

#### Covariates

Our analyses controlled for six demographic factors: age in years, gender, education (0 = less than high school to 6 = PhD or equivalent), annual household income (0 = <$20,000 to 5 = $100,000 or more), race (2 dummy-coded variables representing White, Black, or Other), and Hispanic ethnicity.

### Statistical Approach

For each of the eight preventive behaviors, we conducted separate logistic regressions predicting whether the participants reported engaging in the behavior from their perceived behavioral control, attitudes, and subjective norm of that behavior. All predictors were standardized, and the analyses controlled for all six demographic covariates. We did not use structural equation modeling as some other research has adopted (36, 37) because our focus was on specific single-item behaviors, and single-item measures are not appropriate for latent variables. All our variables of interest are manifest. In a set of follow-up analyses, we examined whether any of the associations were moderated by age in years and whether the three factors interacted in their association with preventive behaviors. Significant interactions were probed using simple slopes at theoretically meaningful values (38). For age, the meaningful values we chose were the mean age for young adults and the mean age for older adults. All materials, data, and code used in this study are available for review online at: https://osf.io/ketfx/?view_only=95cd127049c440a0b5ce1e7e632e586e.

## Results

Descriptive statistics for the full sample and by age categories are provided in Table 1 and Supplementary Table 1. For most preventive behaviors, roughly 70% of study participants reported engaging in the recommended behaviors. There was a higher rate for washing hands (95%) as well as social distancing (82%), but a substantially lower rate for wearing a face mask (19%). There were small age differences in the rate of most preventive behaviors, but older adults were more likely to report social distancing and less likely to report wearing a mask at the time of our assessment.

**Table 1:** Descriptive statistics for full sample and by age category.

	**All participants**	**Younger adults**	**Middle-aged adults**	**Older adults**
*N*	2,256	659	1,056	541
Age range	1898	1839	4064	6598
Age mean	50.17 (16.78)	29.43 (6.86)	52.13 (7.21)	71.62 (4.76)
Female	47.52%	56.90%	48.86%	33.46%
Education	3.16 (1.51)	2.88 (1.56)	3.16 (1.50)	3.48 (1.41)
Income	2.93 (1.76)	2.49 (1.75)	3.05 (1.79)	3.26 (1.60)
Race: white	66.80%	46.28%	69.79%	85.95%
Race: black	15.91%	25.95%	13.73%	7.95%
Race: other	17.29%	27.77%	16.48%	6.10%
Ethnicity: Hispanic	11.48%	20.33%	9.28%	4.99%
**Preventive behaviors**				
Washing hands	94.73%	91.96%	95.64%	96.30%
Using hand sanitizer	71.85%	76.48%	71.88%	66.17%
Not touching your face	71.01%	69.65%	73.39%	68.02%
Social distancing	81.60%	69.95%	84.19%	90.76%
Wearing a face mask	19.24%	30.65%	17.71%	8.32%
Disinfecting surfaces	71.81%	68.74%	73.11%	73.01%
Coughing in your elbow	74.34%	68.59%	75.09%	79.85%
Staying home if sick	68.22%	67.37%	67.80%	70.06%

*Values represent a count, range, percent, or mean (standard deviation). Education was assessed on a scale from 0 = less than high school to 6 = PhD or equivalent. Income was measured on a scale from 0 = <$20,000 to 5 = $100,000 or more.*

### Washing Hands

Higher perceived behavioral control, attitudes, and subjective norm of washing hands were all independently associated with engaging in that behavior (See Table 2). However, the association with perceived behavioral control was substantially stronger than with attitudes or subjective norm. The association for perceived subjective norm (but not behavioral control or attitudes) of washing hands was significantly moderated by age in years (See Table 3), such that it was stronger for the average older adult (OR = 1.94 [1.32, 2.87], *p* < 0.001) than for the average younger adult (OR = 1.20 [0.96, 1.51], *p* = 0.112), which was non-significant. There were no significant interactions between perceived behavioral control, attitudes, and subjective norm in their associations with washing hands (See Table 4).

**Table 2:** Logistic regressions predicting preventive behavior from perceived behavioral control, attitudes, and subjective norm of that behavior.

**Outcomes**	**Predictors**		
	**Control**	**Attitudes**	**Subjective norm**
Washing hands	**1.93 [1.68, 2.22]**, **<0.001**	**1.37 [1.12, 1.68], 0.002**	**1.37 [1.14, 1.65]**, **<0.001**
Using hand sanitizer	**2.38 [2.15, 2.65]**, **<0.001**	**1.19 [1.06, 1.32], 0.002**	**1.33 [1.19, 1.48]**, **<0.001**
Not touching your face	**1.97 [1.77, 2.20]**, **<0.001**	**1.37 [1.24, 1.51]**, **<0.001**	**1.30 [1.17, 1.45]**, **<0.001**
Social distancing	**1.48 [1.33, 1.66]**, **<0.001**	**1.77 [1.59, 1.98]**, **<0.001**	**1.15 [1.03, 1.29], 0.012**
Wearing a face mask	**2.18 [1.90, 2.52]**, **<0.001**	**1.14 [1.02, 1.28], 0.020**	**1.28 [1.14, 1.44]**, **<0.001**
Disinfecting surfaces	**2.18 [1.96, 2.42]**, **<0.001**	**1.33 [1.20, 1.47]**, **<0.001**	**1.26 [1.13, 1.41]**, **<0.001**
Coughing in your elbow	**1.72 [1.56, 1.90]**, **<0.001**	**1.24 [1.12, 1.37]**, **<0.001**	1.08 [0.98, 1.20], 0.119
Staying home if sick	**1.47 [1.34, 1.61]**, **<0.001**	**1.16 [1.06, 1.27], 0.001**	**1.20 [1.09, 1.32]**, **<0.001**

*Values represent standardized odds ratios [95% confidence interval], p-value. All predictors are modeled simultaneously. Each outcome is modeled separately. Models control for demographic covariates. Bolded values are statistically significant = 0.05*.

**Table 3:** Age in years moderating the associations of perceived behavioral control, attitudes, and subjective norm with preventive behaviors.

**Outcomes**	**Predictor moderations**		
	**Age Control**	**Age Attitudes**	**Age Subjective Norm**
Washing hands	0.98 [0.85, 1.12], 0.744	0.98 [0.80, 1.20], 0.838	**1.21 [1.00, 1.46], 0.048**
Using hand sanitizer	**1.14 [1.03, 1.27], 0.013**	1.08 [0.97, 1.19], 0.157	**1.13 [1.01, 1.26], 0.029**
Not touching your face	**1.16 [1.05, 1.29], 0.005**	0.98 [0.89, 1.08], 0.676	1.06 [0.96, 1.18], 0.249
Social distancing	**1.15 [1.03, 1.29], 0.015**	0.92 [0.82, 1.03], 0.134	1.10 [0.98, 1.24], 0.106
Wearing a face mask	**1.22 [1.06, 1.41], 0.007**	**1.13 [1.00, 1.26], 0.045**	**1.14 [1.01, 1.30], 0.031**
Disinfecting surfaces	**1.12 [1.01, 1.24], 0.034**	**0.87 [0.79, 0.96], 0.007**	1.10 [0.99, 1.23], 0.064
Coughing in your elbow	1.06 [0.96, 1.16], 0.232	0.98 [0.89, 1.08], 0.693	1.08 [0.97, 1.19], 0.154
Staying home if sick	1.03 [0.94, 1.14], 0.484	0.94 [0.86, 1.02], 0.140	0.97 [0.88, 1.06], 0.467

*Values represent standardized odds ratios [95% confidence interval], p-value. The moderator age in years was entered as a continuous variable. All predictors are modeled simultaneously. Each outcome is modeled separately. Models control for demographic covariates. Bolded values are statistically significant = 0.05*.

**Table 4:** Interactions between perceived behavioral control, attitudes, and subjective norm predicting preventive behaviors.

**Outcomes**	**Predictor interactions**		
	**Control Attitudes**	**Control Subjective Norm**	**Attitudes Subjective Norm**
Washing hands	1.00 [0.87, 1.15], >0.999	1.05 [0.93, 1.19], 0.403	1.15 [0.98, 1.36], 0.091
Using hand sanitizer	0.94 [0.84, 1.06], 0.343	1.11 [0.99, 1.23], 0.069	1.07 [0.98, 1.18], 0.149
Not touching your face	1.00 [0.90, 1.10], 0.996	0.97 [0.88, 1.08], 0.611	1.00 [0.91, 1.10], 0.945
Social distancing	1.01 [0.90, 1.12], 0.906	0.90 [0.82, 1.00], 0.053	0.95 [0.86, 1.05], 0.314
Wearing a face mask	**0.82 [0.71, 0.95], 0.009**	0.93 [0.81, 1.06], 0.268	1.00 [0.91, 1.10], 0.926
Disinfecting surfaces	1.04 [0.93, 1.16], 0.528	**0.88 [0.79, 0.97], 0.014**	1.02 [0.93, 1.12], 0.639
Coughing in your elbow	1.02 [0.92, 1.13], 0.673	1.01 [0.92, 1.10], 0.841	0.98 [0.90, 1.07], 0.731
Staying home if sick	**0.88 [0.79, 0.97], 0.015**	0.95 [0.87, 1.03], 0.220	0.97 [0.90, 1.06], 0.527

*Values represent standardized odds ratios [95% confidence interval], p-value. All predictors are modeled simultaneously. Each outcome is modeled separately. Models control for demographic covariates. Bolded values are statistically significant = 0.05*.

### Using Hand Sanitizer

Higher perceived behavioral control, attitudes, and subjective norm of using hand sanitizer were all independently associated with engaging in that behavior (See Table 2). However, the association with perceived behavioral control was substantially stronger than with attitudes or subjective norm. The associations for perceived behavioral control and subjective norm of using hand sanitizer were both significantly moderated by age in years (See Table 3), such that they were stronger for the average older adult (Behavioral control: OR = 2.81 [2.37, 3.36], *p* < 0.001; Subjective norm: OR = 1.61 [1.31, 1.97], *p* < 0.001) than for the average younger adult (Behavioral control: OR = 2.00 [1.69, 2.37], *p* < 0.001; Subjective norm: OR = 1.19 [1.03, 1.38], *p* = 0.022), but they both remained significant. There were no significant interactions between perceived behavioral control, attitudes, and subjective norm in their associations with using hand sanitizer (See Table 4).

### Not Touching Your Face

Higher perceived behavioral control, attitudes, and subjective norm of not touching your face were all independently associated with engaging in that behavior (See Table 2). However, the association with perceived behavioral control was substantially stronger than with attitudes or subjective norm. The association for perceived behavioral control of not touching your face was significantly moderated by age in years (See Table 3), such that it was stronger for the average older adult (OR = 2.39 [2.01, 2.87], *p* < 0.001) than for the average younger adult (OR = 1.65 [1.40, 1.94], *p* < 0.001), but remained significant. There were no significant interactions between perceived behavioral control, attitudes, and subjective norm in their associations with not touching your face (See Table 4).

### Social Distancing

Higher perceived behavioral control, attitudes, and subjective norm of social distancing were all independently associated with engaging in that behavior (See Table 2). However, the associations with both perceived behavioral control and attitudes were substantially stronger than with subjective norm. The association for perceived behavioral control of social distancing was significantly moderated by age in years (See Table 3), such that it was stronger for the average older adult (OR = 1.88 [1.51, 2.33], *p* < 0.001) than for the average younger adult (OR = 1.32 [1.13, 1.53], *p* < 0.001), but remained significant. There were no significant interactions between perceived behavioral control, attitudes, and subjective norm in their associations with social distancing (See Table 4).

### Wearing a Face Mask

Higher perceived behavioral control, attitudes, and subjective norm of wearing a face mask were all independently associated with engaging in that behavior (See Table 2). However, the association with perceived behavioral control was substantially stronger than with attitudes or subjective norm. The associations for perceived behavioral control, attitudes, and subjective norm of wearing a face mask were all significantly moderated by age in years (See Table 3), such that they were stronger for the average older adult (Behavioral control: OR = 2.98 [2.25, 3.98], *p* < 0.001; Attitudes: OR = 1.38 [1.10, 1.74], *p* = 0.007; Subjective norm: OR = 1.64 [1.27, 2.11], *p* < 0.001) than for the average younger adult (Behavioral control: OR = 1.80 [1.50, 2.17], *p* < 0.001; Attitudes: OR = 1.02 [0.89, 1.18], *p* = 0.766; Subjective norm: OR = 1.16 [1.01, 1.34], *p* = 0.039). There was a significant negative interaction between perceived behavioral control and attitudes of wearing a face mask, such that the association with behavioral control was smaller among those who believed that wearing a face mask was more effective and the association with attitudes was smaller among those who believed that wearing a face mask was easier (See Table 4).

### Disinfecting Surfaces

Higher perceived behavioral control, attitudes, and subjective norm of disinfecting surfaces were all independently associated with engaging in that behavior (See Table 2). However, the association with perceived behavioral control was substantially stronger than with attitudes or subjective norm. The associations for perceived behavioral control and attitudes of disinfecting surfaces were both significantly moderated by age in years (See Table 3). For perceived behavioral control, the association was stronger for the average older adult (OR = 2.54 [2.13, 3.04], *p* < 0.001) than for the average younger adult (OR = 1.92 [1.65, 2.25], *p* < 0.001), but remained significant. In contrast, for attitudes, the association was stronger for the average younger adult (OR = 1.53 [1.32, 1.78], *p* < 0.001) than for the average older adult (OR = 1.09 [0.92, 1.30], *p* = 0.305), which was non-significant. There was a significant negative interaction between perceived behavioral control and subjective norm of disinfecting surfaces, such that the association with behavioral control was smaller among those who believed that disinfecting surfaces was more normative and the association with subjective norm was smaller among those who believed that disinfecting surfaces was easier (See Table 4).

### Coughing in Your Elbow

Higher perceived behavioral control and attitudes of coughing in your elbow were both independently associated with engaging in that behavior; the association with perceived subjective norm was not significant (See Table 2). The association with perceived behavioral control was substantially stronger than with attitudes. The associations with coughing in your elbow were not significantly moderated by age in years (See Table 3). There were no significant interactions between perceived behavioral control, attitudes, and subjective norm in their associations with coughing in your elbow (See Table 4).

### Staying Home if Sick

Higher perceived behavioral control, attitudes, and subjective norm of staying home if sick were all independently associated with engaging in that behavior (See Table 2). However, the association with perceived behavioral control was substantially stronger than with attitudes or subjective norm. The associations with staying home if sick were not significantly moderated by age in years (See Table 3). There was a significant negative interaction between perceived behavioral control and attitudes of staying home if sick, such that the association with behavioral control was smaller among those who believed that staying home if sick was more effective and the association with attitudes was smaller among those who believed that staying home if sick was easier (See Table 4).

## Discussion

In the present study, we found that higher perceived behavioral control, attitudes, and subjective norm of eight different behaviors that reduce the spread of coronavirus are all independently associated with engaging in those behaviors, which supports the TPB. Across most behaviors, the associations with perceived behavioral control were substantially stronger than with attitudes or subjective norm. Additionally, the associations with perceived behavioral control tended to be stronger among older adults. Also, the three predictors typically did not display significant interactions. There were just a few exceptions to these general findings: subjective norm was not associated with coughing in your elbow, attitudes was more strongly associated with social distancing than perceived behavioral control or subjective norm, associations with perceived behavioral control did not vary by age for three of the outcomes, and there were negative interactions between perceived behavioral control and attitudes or subjective norm for three of the outcomes.

These results demonstrate the importance of individual beliefs like perceived behavioral control, attitudes, and subjective norm in understanding why some people engage in a behavior while others do not, which supports the general hypotheses of the TPB (6). Our findings are in line with previous research on TPB and general health behaviors (13, 15, 16, 18) as well as COVID-19 related preventive behaviors (23, 24, 39, 40). In the context of the ongoing pandemic, these results highlight how the public's beliefs about perceived behavioral control, attitudes, and subjective norm of preventive health behaviors are associated with whether they engage in those behaviors and thereby reduce the spread of coronavirus.

Although the policy of COVID-19 control has changed since March 2020, most U.S. states as well as Washington, D.C. and Puerto Rico still have at least one restrictive measure in place and the CDC has updated its guidelines, recommending to get a vaccine and continue to wear a mask in public (4). Our findings may have implications for how public health organizations design their initiatives (41, 42) to increase the number of people engaging in these behaviors. First, in general, perceived behavioral control, attitudes, and subjective norm were all independently associated with engaging in the preventive behaviors, so it may be beneficial to ensure that initiatives simultaneously target all three factors. Beyond these core components, public health officials may also want to take perceived knowledge of COVID-19 into account. Several COVID-19 studies have shown that the perceived knowledge and understanding of the disease is associated with intentions to engage in preventive behavior (23, 36, 37, 43). More specifically, accurate knowledge of COVID-19 is positively associated with attitudes and subjective norms (36), the actual practice of preventive measures (23), and following governmental lockdown implementations (37). Promoting accurate knowledge across the entire population may be a challenge, but one that affords the potential to increase adherence to precautions. Of note, different sources of information exposure may have different effects on compliance of such precautions: For instance, specific exposure through official web-based media, unofficial web-based media, television, newspapers and magazines were positively associated with hand sanitizing, while a negative association was found for exposure through face-to-face communication (44).

Second, perceived behavioral control typically displayed a much stronger association than attitudes or subjective norm, so it may be useful to devote more resources to emphasizing easier ways to engage in these preventive behaviors or address barriers that make it difficult to adhere to the recommendations. For example, because of economic or work related constrains, it can be difficult for some people to maintain social distancing, washing hands, or avoid going to work if sick. It should be noted, however, that perceived behavioral control does not always have the strongest predictive power among the core TPB components; in other studies, attitudes (37) or subjective norm (36) revealed stronger associations with behavioral intentions.

Third, the association with behavioral control tended to be stronger among older adults, so initiatives targeting older adults should particularly focus on techniques to make these behaviors easier to perform. In contrast, initiatives that specifically target younger adults should aim for more balance between the three factors, perhaps with more educational components about attitudes and more social components showing that their peers engage in the behaviors. It might also be worthwhile to put close family at the core of (governmental) measures in the ongoing pandemic. A German study, for example, reported that their sample with an average age of 27 years distinguished between their close family and other social spheres (wider family and friends, colleagues at work, and society in general), and although all of the social spheres were relevant in the context of the pandemic, the close family showed the highest importance (45).

The few exceptions to the general trends we found might also be useful to take into account when designing initiatives. For example, if the goal is to specifically target the behavior of coughing and sneezing into your elbow or tissues, then it may not be useful to emphasize subjective norm as that factor was not associated. Instead, more emphasis might be put on behavioral control and attitudes for that behavior. Additionally, initiatives targeting social distancing behavior should particularly focus on teaching about attitudes, as that factor displayed the strongest association, and do not need to put as much emphasis on behavioral control, which was not as strongly associated with social distancing as it was with other preventive behaviors. Initiatives should focus on increasing positive attitudes (vs. reducing negative attitudes) since a recent study showed that positive but not negative attitudes (inconvenience and lack of necessity) were significantly associated with the number of close physical contacts, avoidance of social gatherings, and physical distancing in public venues (24). Likewise, it has been recommended to promote a positive attitude toward wearing a face mask to enable people to believe that they can stay in full control of their own health (39). Finally, although it might be beneficial to adjust the relative emphasis of behavioral control depending on the age of the target audience, it may not be useful to do so if the initiative only addresses washing hands, coughing in your elbow, or staying home if sick, as the association with behavioral control was not moderated by age for those behaviors.

Besides providing support for the TPB as a useful framework to predict preventive behaviors during the COVID-19 pandemic, our study also provides a snapshot of the overall level of engagement in those behaviors in a nation-wide sample covering most of the adult lifespan. In general, ~70% of respondents in this study reported engaging in the recommended behaviors, though the percentage for washing hands was higher (95%) and wearing a face mask was substantially lower (19%). This pattern is similar to preventive behavior percentages previously reported for the 201516 influenza season (35), which also found that washing hands was the most commonly reported behavior (83%) and wearing a face mask was one of the least reported (19%). It should be noted, though that our study was conducted before the CDC began to officially recommend the use of face masks in public settings to prevent the spread of coronavirus (i.e., April 3, 2020) (46). The rate of wearing face masks has likely increased in response to changing CDC guidelines. However, as we observed the hypothesized TPB associations in this study for behaviors that vary greatly in reported adherence (1995%), we do not expect that an increase in wearing face masks would substantially alter the TPB associations we found.

With regard to the TPB framework, the three core components typically did not moderate each other, which indicates that the relationship between these factors is not interactive. It might be more appropriate to modify the paths within the TPB framework differently and to add other moderating variables: Several studies suggest a causal effect of subjective norm on attitudes, such that people's perceived social pressure from critical others contributes to increasing their attitudes toward specific behaviors (36, 47, 48). Moreover, Han et al. (36) found that psychological risk moderated the association between subjective norm and attitudes. In their study on US tourists' post-pandemic travel intentions for safer international destinations, they found that US tourists who feel a high psychological risk (i.e., anxiety/stress/discomfort/fear that stems from being a tourist) of traveling to any country seriously affected by COVID-19 are more likely to have a positive attitude toward safer destination choices than those with a low psychological risk. Future studies should also integrate factors like psychological risk and perceived knowledge to extend the theoretical framework of the TPB in other contexts, which will ultimately contribute to a better understanding of human behavior.

### Strengths

The data for this research was collected during the COVID-19 pandemic, allowing for a unique look into individual factors associated with engaging in preventive health behaviors in the midst of a crisis. The sample included participants across the adult lifespan, allowing for a meaningful examination of how the associations vary by age. We separately examined eight specific preventive health behaviors, which enabled our comparison of the similarities and differences in associations for each behavior.

### Limitations and Future Directions

As with any study, some degree of selection bias is possible. We used an online questionnaire to collect data, and it is thus possible that people with limited internet access or low digital literacy were not included in the present sample. It could be that perceived behavioral control, attitudes, subjective norms, and the engagement in preventive behaviors are different for people who do not use the internet. Internet access/use may affect the knowledge about COVID-19 (49), which in turn can influence attitudes and subjective norms (36). Likewise, the sample may not be entirely representative of the US population due to the recruitment process. The study is further limited to the United States and to the early phase of the pandemic. Now, 1 year later, we would expect that these associations still hold based on both the TPB itself and the large literature on the TPB and preventive health behaviors. It seems, however, possible that specific pandemic-related factors such as being vaccinated, having recovered from COVID, or being tired of the pandemic weaken positive attitudes toward preventive behaviors or reduce subjective norms in some people, which in turn might affect engagement in preventive behaviors. Further research may take such variables into account as they could refine our knowledge on the role of the TBP in pandemic-related behavior. Also, the measure of behavior was self-reported and may not be completely accurate. Future studies could apply mobile sensing or the electronically activated recorder (50) to assess preventive behaviors more objectively. We examined the associations with eight different preventive health behaviors, but there are many other preventive behaviors we did not examine that could have different associations. For example, we did not assess getting a COVID-19 vaccination since no vaccine was available at the time of data collection, but for this specific behavior, we would expect similar associations based on other studies (19, 20). Additionally, although this study found associations with perceived behavioral control, attitudes, and subjective norm, further research would be needed to identify the specific reasons and barriers associated with avoiding each of these preventive behaviors. The present study was cross-sectional, so it is not entirely clear whether perceived behavioral control, attitudes, and subjective norm lead to future engagement in preventive health behaviors or whether previously engaging in the behaviors leads to perceptions of behavioral control, attitudes, and subjective norm. Future longitudinal studies and interventions might help to disentangle this directionality question.

## Conclusions

During the COVID-19 pandemic, higher perceived behavioral control, attitudes, and subjective norm of preventive health behaviors were associated with greater likelihood of engaging in those behaviors. These findings are consistent with prior research on the TPB and demonstrate that the associations are present in the midst of a global crisis. This research provides important information for public health organizations as they design initiatives to increase compliance with their behavior recommendations. Specifically, we suggest that they need to address all three factors: perceived behavioral control, attitudes, and perceived subjective norm of the preventive behaviors. However, perceived behavioral control should receive the most emphasis, particularly when targeting older adults. We hope that these suggestions will be useful in reducing the spread of coronavirus and any similar diseases in the future.

## Data Availability Statement

The datasets presented in this study can be found in online repositories. The names of the repository/repositories and accession number(s) can be found below: https://osf.io/ketfx/?view_only=95cd127049c440a0b5ce1e7e632e586e.

## Ethics Statement

The studies involving human participants were reviewed and approved by Institutional Review Board of Florida State University. The patients/participants provided their written informed consent to participate in this study.

## Author Contributions

DA: conceptualization, methodology, writing original draft, and writing revision. JES: conceptualization, methodology, formal analysis, investigation, data curation, writing original draft. AAS, JL, and ML: conceptualization, methodology, and writing review and editing. AT and ARS: conceptualization, methodology, writing review and editing, and supervision. All authors contributed to the article and approved the submitted version.

## Conflict of Interest

The authors declare that the research was conducted in the absence of any commercial or financial relationships that could be construed as a potential conflict of interest.
