# Supplementary material for: Preventive Behaviors During the COVID-19 Pandemic: Associations With Perceived Behavioral Control, Attitudes, and Subjective Norm
Source: Front Public Health. 2021 May 7;9:662835. doi: 10.3389/fpubh.2021.662835 (PMC8139398; doi:10.3389/fpubh.2021.662835)
Supplement: Supplementary file 1 [file Table_1.docx]

Supplementary Table S1

*Additional Descriptive statistics for full sample and by age category.*

|  | All Participants | Younger Adults | Middle-Aged Adults | Older Adults |
| --- | --- | --- | --- | --- |
| *Perceived Control* |  |  |  |  |
| Washing hands | 4.61(0.72) | 4.54(0.79) | 4.62(0.69) | 4.66(0.66) |
| Using hand sanitizer | 4.28(1.02) | 4.32(0.94) | 4.28(1.04) | 4.21(1.06) |
| Not touching your face | 3.38(1.29) | 3.55(1.29) | 3.37(1.29) | 3.21(1.28) |
| Social distancing | 4.00(1.06) | 3.88(1.16) | 3.98(1.06) | 4.20(0.89) |
| Wearing a face mask | 3.34(1.30) | 3.61(1.27) | 3.29(1.31) | 3.10(1.23) |
| Disinfecting surfaces | 4.26(0.95) | 4.16(1.03) | 4.32(0.90) | 4.26(0.92) |
| Coughing in your elbow | 4.45(0.85) | 4.33(0.97) | 4.49(0.80) | 4.53(0.75) |
| Staying home if sick | 4.41(0.96) | 4.18(1.09) | 4.39(0.95) | 4.70(0.68) |
| *Attitudes* |  |  |  |  |
| Washing hands | 3.92(1.41) | 3.62(1.53) | 4.01(1.34) | 4.08(1.32) |
| Using hand sanitizer | 3.76(1.32) | 3.51(1.42) | 3.84(1.26) | 3.88(1.26) |
| Not touching your face | 3.82(1.32) | 3.59(1.40) | 3.91(1.27) | 3.91(1.31) |
| Social distancing | 3.83(1.35) | 3.57(1.41) | 3.93(1.28) | 3.97(1.34) |
| Wearing a face mask | 3.24(1.26) | 3.31(1.33) | 3.27(1.28) | 3.13(1.14) |
| Disinfecting surfaces | 3.88(1.31) | 3.67(1.39) | 3.96(1.25) | 3.98(1.28) |
| Coughing in your elbow | 3.79(1.32) | 3.55(1.40) | 3.86(1.25) | 3.93(1.33) |
| Staying home if sick | 4.04(1.39) | 3.75(1.45) | 4.14(1.33) | 4.21(1.40) |
| *Subjective Norm* |  |  |  |  |
| Washing hands | 3.59(0.90) | 3.58(1.13) | 3.58(0.86) | 3.62(0.63) |
| Using hand sanitizer | 3.50(0.87) | 3.62(1.02) | 3.47(0.85) | 3.40(0.67) |
| Not touching your face | 3.19(0.95) | 3.32(1.07) | 3.18(0.93) | 3.04(0.78) |
| Social distancing | 3.34(0.94) | 3.41(1.13) | 3.31(0.90) | 3.31(0.73) |
| Wearing a face mask | 2.84(1.05) | 3.25(1.16) | 2.73(1.01) | 2.56(0.79) |
| Disinfecting surfaces | 3.38(0.95) | 3.52(1.10) | 3.37(0.92) | 3.23(0.77) |
| Coughing in your elbow | 3.36(0.94) | 3.47(1.11) | 3.30(0.92) | 3.32(0.72) |
| Staying home if sick | 3.42(0.95) | 3.49(1.11) | 3.34(0.92) | 3.48(0.78) |

*Note.* Values represent a mean (standard deviation).
